# Patients with Cancer and Immune Checkpoint Inhibitor-Induced Rheumatic irAEs Treated by DMARDs: The RHUMICI Single-Centre Retrospective Cohort

**DOI:** 10.3390/cancers18142213

**Published:** 2026-07-09

**Authors:** Emmanuel Massy, Julien Seiller, Muriel Piperno, Edith Bonnelye, Denis Maillet, Stéphane Dalle, Clara Fontaine-Delaruelle, Pierre-Jean Souquet, Julien Péron, Cyrille B. Confavreux

**Affiliations:** 1 Centre Expert des Métastases Osseuses (CEMOS)-Service de Rhumatologie Sud, Hôpital Lyon Sud, Institut de Cancérologie des Hospices Civils de Lyon (IC-HCL), F-69310 Pierre-Bénite, Francemuriel.piperno@chu-lyon.fr (M.P.);; 2 Université Claude Bernard Lyon 1, F-69003 Lyon, France; 3 Centre de Recherche en Cancerologie de Lyon (CRCL)-UMR INSERM 1052 CNRS 5286, F-69373 Lyon, France; 4 CNRS, Inserm, CHU Lille, UMR9020-U1277-CANTHER-Cancer Heterogeneity Plasticity and Resistance to Therapies, University of Lille, F-59000 Lille, France; 5 Service d’Oncologie Médicale, Institut de Cancérologie des Hospices Civils de Lyon (IC-HCL), F-69310 Pierre-Bénite, France; 6 Service de Dermatologie, Hôpital Lyon Sud, Hospices Civils de Lyon, F-69310 Pierre-Bénite, France; 7 Service de Pneumologie, Hôpital Lyon Sud, Hospices Civils de Lyon, F-69310 Pierre-Bénite, France; 8 INSERM U1290-Research on Healthcare Performance (RESHAPE), F-69008 Lyon, France

**Keywords:** immune checkpoint inhibitors, immune-related adverse events, inflammatory arthritis, disease-modifying antirheumatic drugs, glucocorticoids, oncological outcomes, overall survival, progression-free survival, onco-rheumatology, real-world study

## Abstract

Background/Objectives: Immune checkpoint inhibitors (ICIs) can lead to immune-related adverse events (irAEs), including rheumatic manifestations affecting 5–10% of treated patients. Managing rheumatic irAEs is challenging, as the use of glucocorticoids (GCs) or disease-modifying antirheumatic drugs (DMARDs) raises concerns about potentially impairing ICI antitumour efficacy. The oncological safety of DMARDs used specifically for rheumatic irAE management remains poorly characterised. Our objective was to evaluate the oncological safety profile of various rheumatic irAE treatment strategies. Methods: This single-centre retrospective observational study included 55 patients out of 104 who underwent rheumatological evaluation between July 2016 and October 2022. Patients were categorised into three groups: symptomatic treatment alone (analgesics/NSAIDs, *n* = 11), systemic glucocorticoids (GCs) only (*n* = 27), and conventional synthetic or biological disease-modifying antirheumatic drugs (csDMARDs and/or bDMARDs, *n* = 17). Overall survival (OS) and progression-free survival (PFS) were compared using a Kaplan–Meier analysis. Results: The three most frequent rheumatic irAE patterns were undifferentiated arthritis (*n* = 20), rheumatoid arthritis-like presentations (*n* = 14), and polymyalgia rheumatica (*n* = 11). Patients in the DMARD group had more severe irAEs (CTCAE grade ≥3: 30% vs. 15%, *p* < 0.01) and a higher baseline CRP (27.5 vs. 17.4 mg/L, *p* < 0.05), reflecting greater disease burden at treatment initiation. No significant difference was observed between groups for OS (*p* = 0.22) or PFS (*p* = 0.31) over a median follow-up of 34 months. Conclusions: No detrimental oncological safety signal was identified across rheumatic irAE treatment strategies, including GCs combined with csDMARDs or bDMARDs. These results support the cautious use of DMARDs as GC-sparing agents when clinically indicated, consistent with current EULAR and ESMO guidelines, and underline the need for prospective trials to optimise immunosuppressive management in ICI-treated patients.

## Simple Summary

Immune checkpoint inhibitors have transformed cancer treatment over the past decade by harnessing the immune system to attack tumour cells. This approach comes with a drawback: the same immune activation can cause unintended inflammation in healthy tissues, including joints. These joint side effects occur in up to one in ten patients and often require treatment with cortisone or drugs borrowed from rheumatology practice. A longstanding worry is that dampening the immune response to control these side effects might also blunt the anti-cancer effect of the treatment. To address this, we studied 55 patients who developed joint side effects during checkpoint inhibitor therapy and tracked their cancer outcomes over nearly three years, depending on whether they received cortisone alone or in combination with rheumatology drugs. Cancer outcomes were similar across all treatment groups, suggesting that rheumatology drugs can be added when cortisone is insufficient without compromising the benefit of cancer treatment.

## 1. Introduction

During the past decade, immune checkpoint inhibitors (ICIs) have transformed the management of numerous patients with cancer [1]. Through T-cell activation by targeting regulators such as programmed cell death(-ligand) PD1/PDL1 or cytotoxic T-lymphocyte-associated protein 4 (CTLA4), these treatments stimulate the immune system to induce the antitumor response [2]. However, the activation of the immune system led to the development of a new spectrum of immune-related adverse events (irAEs) that oncologists must take into account [1]. Rheumatic irAEs affect 5% to 10% of patients treated with ICIs, predominantly manifesting as inflammatory arthritis, symmetric polyarthritis, polymyalgia-like syndrome, myositis, and sicca syndrome [3,4]. Of note, rheumatic irAEs may persist after ICI cessation [5], and clinical practice guidelines have therefore been developed to guide the management of these patients [6,7].

The management of irAEs can be challenging, since the use of immunosuppressive treatments raises concerns about potentially compromising the antitumor activity of ICIs, though the magnitude of this effect remains debated, particularly when these agents are used specifically for irAE management [8]. While systemic glucocorticoids (GCs) are used as the first-line therapy following a symptomatic treatment, data suggest they may reduce the antitumor activity of ICIs when used for cancer-related indications or at baseline [9,10,11], while their use specifically for irAE management appears to carry a more favourable oncological risk profile [12]. Conventional synthetic disease-modifying antirheumatic drugs (cs-DMARDs) have been proposed as sparing agents for GCs, and biological (b-) DMARDs are recommended in case of an inadequate response to cs-DMARDs [6]. More recently, the 2024 EULAR points to consider for targeted therapies in patients with inflammatory arthritis and a history of cancer have highlighted the specific need for evidence on the impact of DMARDs in ICI-induced arthritis in the context of active malignancy [13]. However, there are currently limited data regarding the safety of DMARDs regarding ICI effects and tumour response. Previous studies reported the delicate question of the optimization of DMARD administration [4,14]. Therefore, the objectives of the present study were: (1) to describe the characteristics of a cohort of patients with rheumatic irAEs treated by ICIs and (2) to evaluate the oncological safety profile of various rheumatic irAE treatment strategies in terms of OS and PFS.

## 2. Materials and Methods

The RHUMICI study is a single-centre, retrospective, and observational study conducted at the Lyon Sud University Hospital (*Hospices Civils de Lyon*, France) that included adult patients who received ICIs for the treatment of a solid or blood tumour and presented with rheumatic irAEs between July 2016 and October 2022. Patients with a history of active inflammatory rheumatic disease and patients for whom rheumatic irAEs were not assessed by a rheumatologist were excluded. The study was approved by the institutional review board of the *Hospices Civils de Lyon* (N° 22_452 on 13 June 2022) and the *Commission Nationale de l’Informatique et des Libertés* (CNIL, French data protection authority, N° 22_5452—8 July 2022).

### 2.1. Data Collection

Data were extracted from patients’ medical records using a standardized data collection form. Patient characteristics included age, sex, weight, height, Body Mass Index (BMI), smoking habits, as well as history of cardiovascular, rheumatological, and autoimmune conditions. Cancer characteristics encompassed tumour type, metastatic status at inclusion, date of diagnosis, type of ICI used, date of ICI initiation and cessation, reason for ICI cessation, associated treatments, initial tumour response, date of progression, and date of death. The progression was evaluated by the patient’s treating oncologist. Rheumatological characteristics included the presence of auto-antibodies at ICI initiation, time before rheumatic irAE onset, Eastern Cooperative Oncology Group performance status (ECOG-PS), clinical symptoms, and rheumatological patterns of irAEs. Based on standard disease classification [15], the rheumatological patterns of irAEs were determined by the patient’s rheumatologist, who then performed the choice of the administered treatment according to international guidelines [6]. The severity of irAEs was classified according to the Common Terminology Criteria for Adverse Events 5.0 (CTCAE) [16]. Treatment characteristics encompassed the use of symptomatic treatment, initiation and cessation dates of GCs, initiation and cessation dates of cs or b-DMARDs, and clinical improvement after treatment. To describe patients’ characteristics and assess the tumour response, patients were divided into three groups based on the treatment for their rheumatic irAEs: the symptomatic treatment group was composed of patients who received only analgesics or non-steroidal anti-inflammatory drugs (NSAIDs) (*n* = 11); the GC group was composed of patients treated solely with systemic (oral or intravenous) glucocorticosteroids (*n* = 27); and the DMARD group was composed of patients treated with csDMARDs and/or bDMARDs (*n* = 17). Patients who received sequential treatment escalation during follow-up were assigned to the group corresponding to the most intensive therapy ultimately received; the time elapsed under prior, less intensive treatments was therefore attributed to the final treatment group, which may introduce immortal time bias in survival analyses.

### 2.2. Statistical Analysis

The baseline characteristics of the patients are expressed as counts and percentages for categorical variables and as the median and range for continuous variables. For continuous variables, the Kruskal–Wallis test was used to perform multiple comparisons, while the Wilcoxon test was employed to compare two groups. The Fisher’s exact test was used to compare categorical variables. A survival analysis was conducted using the Kaplan–Meier method, and log-rank tests were used for statistical comparisons. All analyses were performed using the R software, version 4.2.1 (©2017; R Foundation for Statistical Computing, Vienna, Austria; https://www.r-project.org/).

## 3. Results

### 3.1. Population

**Figure 1:**
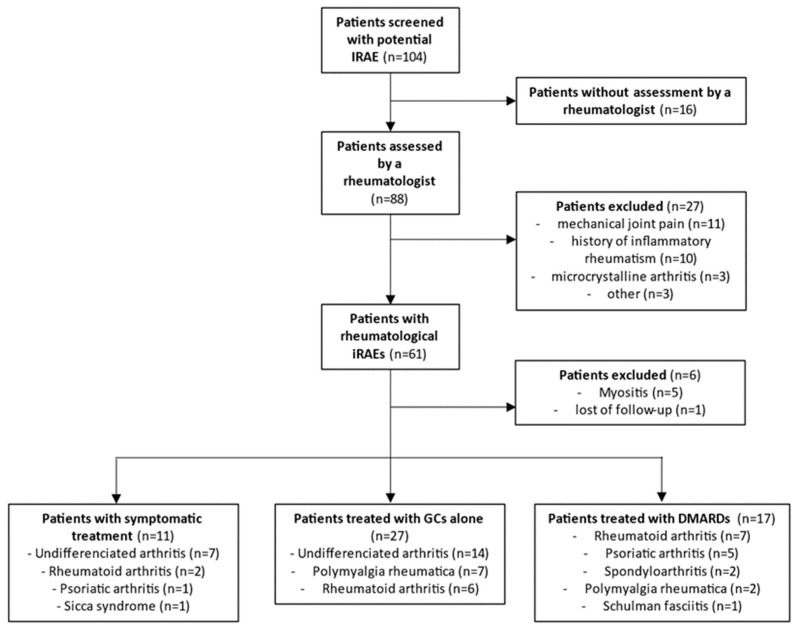
Population flow chart.

A total of 104 patients with suspected irAEs were referred to the rheumatology department and screened. Among them, 16 were excluded due to the absence of assessment by a rheumatologist and 33 since they presented mechanical joint pain (*n* = 11); a history of inflammatory rheumatism (*n* = 10), gout or pseudogout arthritis (*n* = 3), myositis (*n* = 5), or fracture (*n* = 3); or were lost to follow up (*n* = 1) (Figure 1). Consequently, 55 patients were included (Table 1).

The symptomatic treatment group was composed of 11 (20%) patients, the GC-only group was composed of 27 (49%), and the DMARD group was composed of 17 (31%). No significant difference was found between groups regarding patients’ non-oncological history. Although it did not reach statistical significance, there was a trend toward a greater proportion of patients treated for lung cancer in the symptomatic treatment and DMARD groups, as compared to the GC-only group (*p* = 0.09). In the GC-only group, melanoma was predominant. In addition, there was no difference between the groups regarding ICI treatment. The follow-up duration and the time between ICI initiation and rheumatic irAE onset were not significantly different between groups (Table 1 and Table 2).

In the DMARD group, 16 (94%) patients were treated using methotrexate (median dose [IQR]: 15 mg [12.5–17.5]). Seven patients (41%) received bDMARDs, alone or combined with csDMARD: 5 infliximab and 2 tocilizumab. None of them received interleukin-17 (IL-17) inhibitors, interleukin-23 (IL-23) inhibitors, anti-CD20 or Janus kinase (JAK) inhibitors.

### 3.2. Characteristics of Rheumatic irAEs

The detailed patterns of rheumatic and non-rheumatic irAEs are presented in Table 2. The rheumatic irAE pattern differed between groups; there were more patients with defined rheumatic diseases in the DMARD group compared to the GC-only and symptomatic treatment groups, in which there was a greater proportion of patients with undifferentiated arthritis (*p* < 0.0001). Patients in the DMARD group had a higher CTCAE grade; no patient had >2 in the symptomatic treatment group, 4 (15%) in the GC-only group, and 5 (30%) in the DMARD group (*p* < 0.01). At rheumatic irAE onset, patients in the DMARD group had higher CRP serum levels (the median was 27.5 mg/L compared to 17.4 mg/L in the GC-only group and 7.0 mg/L in the symptomatic treatment group; *p* < 0.05).

Regarding treatment strategy, 14 (82%) patients in the DMARD group also received corticosteroids but at a numerically lower dose than in the GC-only group; the median initial dose was 15 mg/day in the DMARD group compared to 20 mg/day in the GC-only group. No significant difference was found regarding the duration of the corticosteroid treatment between both groups (*p* = 0.09); most patients were treated for a period longer than 3 months.

### 3.3. The Impact of the Rheumatic irAE Treatment on Oncological Outcomes

**Figure 2:**
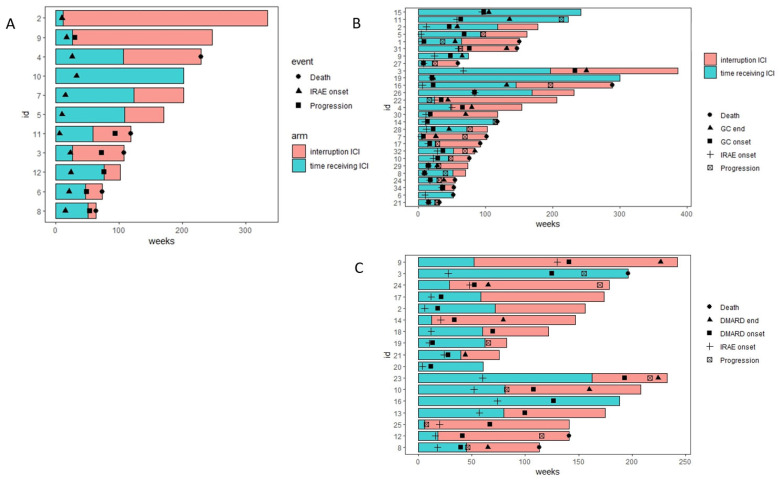
Swimmer plot of the symptomatic, GC and DMARD groups. (**A**) Symptomatic group. (**B**) GC group with patients receiving GC initial dose of <10 mg/day (upper section) or >10 mg/day (bottom section). (**C**) DMARD group with patients receiving csDMARDs (upper section) or bDMARDs (bottom section).

Events occurring after ICI initiation are visually represented in a swimmer plot (Figure 2). Cancer progression occurred in 40 patients: 6 (55%) in the symptomatic treatment group (Figure 2A), 17 (63%) in the GC-only group (Figure 2B), and 8 (47%) in the DMARD group, comprising 5 of the 7 patients who received bDMARD (Figure 2C). The progression predominantly occurred after the onset of rheumatic irAEs. In the swimmer plot for the GC-only group, 8 patients (top section) were treated with low-dose prednisolone (<10 mg/day) at irAE onset, while 19 patients (bottom section) received higher doses (>10 mg/day; Figure 2B). Disease progression occurred in 5 of the 8 patients (62.5%) in the low-dose group compared to 13 of the 19 patients (68.4%) in the high-dose group.

**Figure 3:**
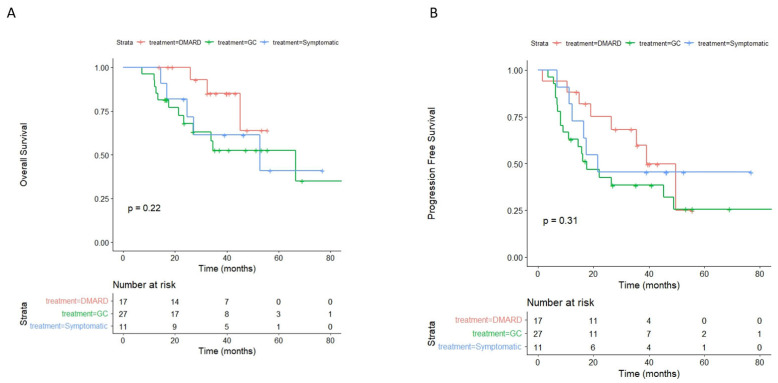
Kaplan–Meier plots for (**A**) Overall Survival and (**B**) Progression Free Survival for types of rheumatological irAE treatment. Corresponding risk tables are outlined below the plots.

Overall survival did not differ significantly between the three treatment groups (log-rank *p* = 0.22; Figure 3A). In the GC-only group, the median OS was 35 months, and 55 months in the symptomatic treatment group (HR = 0.78, 95%CI 0.28–2.16, *p* = 0.63), while the median OS was not reached in the DMARD group (HR = 0.55, 95%CI 0.21–1.45, *p* = 0.23). Progression-free survival similarly did not differ significantly between groups (log-rank *p* = 0.31; Figure 3B), with a median PFS of 16 months in the GC-only group, 21 months in the symptomatic treatment group (HR = 0.65, 95%CI 0.28–1.53, *p* = 0.33), and 40 months in the DMARD group (HR = 0.73, 95%CI 0.36–1.45, *p* = 0.36). OS and PFS did not differ between patients who received csDMARD only (*n* = 10) and those who received bDMARDs (as a monotherapy or combined, *n* = 7).

## 4. Discussion

The present RHUMICI study that included 104 patients referred to the rheumatology department for suspected irAEs reported that half of these patients were diagnosed with confirmed rheumatic irAEs. Three predominant clinical patterns emerged in the cohort: undifferentiated arthritis (20 cases), rheumatoid arthritis-like presentations (14 cases), and polymyalgia rheumatica (11 cases). The less frequent presentations were psoriatic arthritis (6 cases), spondyloarthritis (2 cases), Schulman fasciitis (1 case), and Sicca syndrome (1 case).

To evaluate the impact of immunosuppressive therapies on oncological outcomes, patients were categorised into three groups based on treatment type: symptomatic management alone, glucocorticoids (GCs) only, or GCs combined with disease-modifying antirheumatic drugs (DMARDs). No statistically significant difference in overall survival (OS) or progression-free survival (PFS) was observed between groups. These findings are consistent with an absence of a negative oncological safety signal from the use of DMARDs in the context of rheumatic irAEs and should be interpreted as hypothesis-generating rather than definitive proof of equivalence, given the inherent limitations of the retrospective design and the sample size. Consistent with findings from other cohorts, approximately half of the patients referred for rheumatologic assessment did not present with true rheumatic irAEs [17]. This underscores the importance of systematic rheumatological evaluation when irAEs are suspected, as recommended by both EULAR and ESMO guidelines [6,7]. Moreover, the cohort herein presented the three main entities of rheumatic irAEs most commonly described in the literature [18,19].

Patients escalated to DMARDs presented with more severe rheumatic irAEs at baseline, as reflected by higher CTCAE grades and higher CRP levels. This confounding by indication is an unavoidable limitation of retrospective observational data and precludes any causal interpretation of between-group differences. Yet, paradoxically, cancer progression rates were numerically lower in the DMARD group (47% vs. 63% in the GC-only group). This observation deserves attention: the subgroup carrying the greatest rheumatological burden, and receiving the most intensive immunosuppressive regimen, did not fare worse oncologically. If anything, this pattern lends additional support to the safety of DMARD use in this setting, rather than undermining it.

Melanoma was more frequently represented in the GC-only group. Given the well-established sensitivity of melanoma to ICIs, this imbalance might be expected to favour the GC-only group in terms of oncological outcomes. The fact that OS and PFS remained comparable across groups, despite this distributional advantage, therefore implies that DMARD-treated patients achieved equivalent survival with a baseline tumour profile that was, if anything, less favourable. The melanoma imbalance thus does not confound the main finding; rather, it corroborates it.

Regarding the substantial overlap between groups (with 82% of DMARD-treated patients also receiving concomitant GCs) this reflects routine clinical practice, where treatment escalation (adding a DMARD for GC resistance or GC dependence) is the standard approach rather than treatment substitution. The comparison should therefore be interpreted as GC alone versus GC combined with DMARDs, which is precisely the clinically relevant question faced by rheumatologists and oncologists in daily multidisciplinary management. The median initial GC dose was numerically lower in the DMARD group (15 mg/day) versus the GC-only group (20 mg/day), consistent with the role of DMARDs as GC-sparing agents, as recently demonstrated by Hysa et al. in a pilot study of methotrexate use in ICI-induced arthritis [20].

The oncological outcomes were also evaluated according to GC dose within the GC-only group. Among patients who received low-dose prednisolone (<10 mg/day), 5 of 8 (62.5%) experienced cancer progression, compared to 13 of 19 (68.4%) in the high-dose group (>10 mg/day), suggesting that cancer progression did not appear related to GC dose in this cohort. This is consistent with recent meta-analytic data showing that GCs prescribed specifically for irAEs do not negatively impact survival outcomes, in contrast with GCs prescribed for cancer-related symptoms or at baseline [12,21]. A 2024 systematic review and meta-analysis including 6148 patients with renal cell carcinoma and urothelial carcinoma similarly found that systemic GC use for irAEs had no influence on clinical outcomes [22].

Herein, 7 patients were treated with bDMARDs: 5 received infliximab and 2 tocilizumab. While concerns have been raised regarding the bDMARDs potential to promote cancer progression [23,24], data remain inconclusive. Bass et al. found that TNF inhibitors and tocilizumab were associated with reduced PFS compared to methotrexate after a median follow-up of 1009 days, even among patients with melanoma [24]. However, bDMARDs also represent a potential way to improve ICI efficacy [25]. The 2024 EULAR points to consider for the initiation of targeted therapies in patients with inflammatory arthritis and a history of cancer notably highlighted the need for further studies specifically evaluating the impact of targeted therapies on ICI-induced inflammatory arthritis in the context of active cancer [13]. Emerging data from early-phase clinical trials continue to suggest that TNF inhibitors may potentiate rather than blunt the antitumour effects of ICIs, particularly in melanoma [26,27,28]. Similarly, tocilizumab has been used to treat rheumatic irAEs without negatively impacting oncologic outcomes in open-labelled studies and case series [29,30].

From a regulatory and normative standpoint, the 2024 EULAR points to consider represent the most recent institutional framework for targeted therapies in patients with inflammatory arthritis and malignancy [13]. The accompanying systematic literature review confirmed that targeted therapies were not associated with an increased risk of incident cancer or cancer recurrence compared to csDMARDs [31], providing additional reassurance for the clinical use of these agents in appropriately selected patients.

The present study has some limitations. Due to its single-center design, it may be subject to selection bias, and the relatively small sample size may limit the statistical power, particularly in subgroup analyses. Additionally, variability in cancer types across patient groups could introduce confounding factors. The proportion of male patients was balanced across treatment groups (symptomatic: 55%, GC: 55%, DMARD: 47%, *p* = 0.93), suggesting no significant sex imbalance. However, a formal stratified survival analysis by sex was not feasible given the small sample size and was therefore not performed. Finally, treatment group assignment was based on the most intensive therapy received during the entire follow-up period. Patients who were escalated sequentially (from symptomatic treatment to GC, or from GC to DMARD) were classified according to their final treatment level. This approach carries a risk of immortal time bias, as the period preceding treatment escalation is attributed to the more intensive group, during which, by definition, the patient had not yet experienced the outcome. This may artificially favour the DMARD group in terms of survival. While this limitation applies to most retrospective cohorts addressing treatment escalation strategies, it should be considered when interpreting the between-group survival comparisons. However, the study also presents notable strengths. We specifically examined treatments initiated for rheumatic irAEs, excluding DMARDs started for non-rheumatologic indications such as immune-related colitis. This real-world and observational design allowed for comprehensive data collection encompassing a long median follow-up of 34 months (1029 days), complemented by detailed clinical data, including swimmer plots.

## 5. Conclusions

The present findings suggest the absence of a negative safety signal regarding oncological outcomes in patients with rheumatic irAEs treated with various immunosuppressive strategies, including GCs alone or in combination with csDMARDs or bDMARDs. Rather than concluding equivalence between treatment types, these results support the cautious use of DMARDs as GC-sparing agents when clinically indicated, consistent with current EULAR and ESMO recommendations. These data provide clinically relevant information for multidisciplinary onco-rheumatology teams and underline the urgent need for prospective trials and dedicated registries to optimise treatment strategies in this growing patient population.

## Figures and Tables

**Table 1:** Patients’ characteristics. Results are presented with effective and percentage *n* (%) or median and interquartile range. Statistical tests were performed comparing medians or frequencies between the 3 groups using Kruskal–Wallis or Fisher’s exact tests.

		Overall (*n* = 55)	Symptomatic Group (*n* = 11)	Glucocorticoid Group (*n* = 27)	DMARD Group (*n* = 17)	*p*-Value
Age (years), median (IQR)		70 (60–76)	62 (59–69)	72 (64–77)	70 (61–76)	0.21
Male (%)		29 (53%)	6 (55%)	15 (55%)	8 (47%)	0.93
Weight (Kg), median (IQR)		70 (62–83)	64 (61–74)	73 (64–85)	72 (63–85)	0.62
Height (cm), median (IQR)		165 (159–173)	165 (161–177)	165 (159–174)	167 (161–171)	0.64
BMI (Kg/m^2^), median (IQR)		25 (23.2–28.6)	24.1 (21.8–25.1)	26.2 (23.6–29.2)	24.8 (23.1–29.4)	0.36
Smoking status (*n*)	Current	18	8	5	5	<0.05
	Cessation > 3 years	14		9	5	
	No smoking	23	3	13	7	
Alcohol status (*n*)	Current	2		1	1	0.46
	Cessation	5	1	1	3	
	No alcohol	48	10	25	13	
Cardiovascular history, *n* (%)		29 (53%)	3 (27%)	18 (66%)	8 (47%)	0.09
Rheumatological history, *n* (%)		16 (29%)	3 (27%)	9 (33%)	4 (23%)	0.92
	Microcrystalline rheumatism	6		5	1	
	Osteoarthritis	9	2	4	3	
	Sarcoidosis	1	1			
Auto-immune history, *n* (%)		13 (29%)	3 (27%)	5 (19%)	5 (29%)	0.64
	Chronic lymphocytic thyroiditis	5		3	2	
	Graves’ disease	3	1	2		
	Psoriasis	5	2		3	
Cancer type, *n* (%)	Melanoma	23 (42%)		14 (52%)	5 (29%)	0.09
	Lung cancer	22 (40%)		9 (33%)	6 (35%)	
	Renal cell carcinoma	7 (13%)	3 (27%)	2 (7%)	5 (29%)	
	Urothelial cancer	1 (2%)	8 (73%)	1 (4%)		
	Ovarian cancer	1 (2%)		1 (4%)		
	Hodgkin lymphoma	1 (2%)			1 (6%)	
ICI type, *n* (%)	Nivolumab	30 (55%)		14 (52%)	11 (65%)	0.85
	Pembrolizumab	17 (31%)	5 (45%)	8 (30%)	4 (23%)	
	Nivolumab plus Ipilimumab	4 (7%)	5 (45%)	2 (7%)	1 (6%)	
	Atezolizumab	2 (4%)	1 (9%)	2 (7%)		
	Durvalumab	1 (2%)			1 (6%)	
	Tislelizumab	1 (2%)		1 (4%)		
ICI maintenance at inclusion, *n* (%)		38 (69%)	9 (82%)	20 (74%)	9 (53%)	0.25
ICI treatment duration (months), median (IQR)		14 (6–25)	13 (8–25)	9 (5–16)	14 (9–18)	0.92
Cause of ICI interruption during follow up, *n* (%)	Overall	45/55		22	14	0.42
	Progressive disease	22 (49%)	9	13 (59%)	5 (29%)	
	Complete response	7 (16%)	4 (36%)	3 (14%)	1 (6%)	
	Non-rheumatological irAE	7 (16%)	3 (27%)	2 (8%)	3 (18%)	
	Rheumatological irAE	3 (7%)	2 (18%)	1 (4%)	2 (12%)	
	End of adjuvant treatment	6 (13%)		3 (14%)	3 (18%)	
Associated previous treatment, *n* (%)		19 (35%)	3 (27%)	6 (22%)	10 (59%)	0.16
	Surgery	6 (11%)		2 (7%)	4 (24%)	
	Radiotherapy	8 (15%)	2(18%)	3 (11%)	3 (18%)	
	Chemotherapy	5 (9%)	1 (9%)	1 (4%)	3 (18%)	
Cancer response at inclusion, *n* (%)	Complete response	14 (25%)	2 (18%)	5 (19%)	7 (41%)	0.26
	Partial response	16 (29%)	5 (45%)	7 (26%)	4 (24%)	
	Stable disease	8 (15%)	1 (9%)	3 (11%)	4 (24%)	
	Progressive disease	14 (25%)	3 (27%)	10 (37%)	1 (6%)	
Follow-up duration (months)		34 (18–46)	39 (24–50)	27 (17–44)	36 (28–43)	0.34

**Table 2:** Characteristics of the rheumatologic irAEs and their management. Results are presented with effective and percentage *n* (%) or median and interquartile range. Statistical tests were performed comparing medians or frequencies between the 3 groups using Kruskal–Wallis or Fisher’s exact tests.

		Overall (*n* = 55)	Symptomatic Group (*n* = 11)	Glucocorticoid Group (*n* = 27)	DMARD Group (*n* = 17)	*p*-Value
Weeks before irAE onset, median (IQR)		18 (10–30)	17 (12–23)	16 (10–32)	21 (12–52)	0.52
ECOG status at IRAE onset	0	11 (20%)	2 (18%)	6 (22%)	3 (18%)	0.93
	1	26 (47%)	5 (45%)	12 (44%)	9 (53%)	
	2	4 (7%)	1 (9%)	1 (4%)	2 (12%)	
CRP at inclusion (mg/L), median (IQR)		18.2 (7.5–39.7)	7.0 (1.8–12.7)	17.4 (6.3–31.1)	27.5 (15.4–49.8)	<0.05
Tender joint count, median (IQR)		6 (4–7)	4 (3–6)	5 (4–6)	5 (4–7)	0.56
Rheumatological irAE pattern	Rheumatoid arthritis	14 (26%)	2 (18%)			<0.0001
	Undifferentiated arthritis	20 (36%)	7 (64%)		7 (41%)	
	Polymyalgia rheumatica	11 (20%)				
	Psoriatic arthritis	6 (11%)	1 (9%)	5 (18%)	2 (12%)	
	Spondyloarthritis	2 (4%)		13 (48%)	5 (29%)	
	Schulman fasciitis	1 (2%)		9 (33%)	2 (12%)	
	Sicca syndrome	1 (2%)	1 (9%)		1 (6%)	
Rheumatological irAE CTCAE grade, *n* (%)	1	17 (31%)		8 (30%)	1 (6%)	<0.01
	2	27 (49%)	8 (73%)	13 (48%)	11 (65%)	
	3	8 (15%)	3 (27%)	4 (15%)	4 (24%)	
	4	1 (2%)			1 (6%)	
Non-rheumatological irAE, *n* (%)		36	7	17	12	
	Thyroiditis	11 (31%)	1 (14%)	7 (41%)	3 (25%)	
	Psoriasis	6 (17%)	1 (14%)	2 (12%)	3 (25%)	
	Vitiligo	3 (8%)		2 (12%)	1 (8%)	
	Colitis	2 (6%)	1 (14%)		1 (8%)	
	Rash	3 (8%)		1 (6%)	2 (17%)	
	Diabetes	1 (3%)		1 (6%)		
	Hypophysitis	1 (3%)	1 (14%)			
	Pancreatitis	1 (3%)			1 (8%)	
	Adrenal insufficiency	1 (3%)		1 (6%)		
		1 (3%)		1 (6%)		
	Hepatitis	3 (8%)	1 (14%)	1 (6%)	1 (8%)	
	Uveitis	2 (6%)	1 (14%)	1 (6%)		
	Lung disease	1 (3%)	1 (14%)			
	Nephritis					
Patients with non-rheumatological irAE, *n* (%)		34 (62%)	6 (55%)	16 (57%)	12 (70%)	
	Cumulating 2	5 (10%)	1 (9%)	2 (11%)	2 (12%)	
	Cumulating 3	1 (2%)		1 (7%)		
csDMARD type	Methotrexate				16 (94%)	
Methotrexate dose (mg), median (IQR)					15 (12.5–17.5)	
bDMARD type	Infliximab				5 (29%)	
	Tocilizumab				2 (12%)	
bDMARD history	Sequential treatment				3	
	Combined cs + bDMARDs				3	
	bDMARDs started directly				1	
GC use		41 (75%)		27 (100%)	14 (82%)	
Initial GC dose (mg/d), median (IQR)		15 (0–20)		20 (15–25)	15 (10–20)	0.06
GC duration	>3 months	32 (58%)		20 (74%)		0.09
	7 days to 3 months	6 (11%)		4 (15%)	12 (71%)	
	<7 days	3 (6%)		3 (11%)	2 (12%)	
Time between irAE and GC/DMARD onset (weeks), median (IQR)				1 (0–3)	5 (2–12)	<0.001

## Data Availability

The data supporting the findings of this study are not publicly available due to ethical and privacy restrictions related to patient data protection, in accordance with the approval of the institutional review board of the Hospices Civils de Lyon (N° 22_452) and the Commission Nationale de l’Informatique et des Libertés (CNIL, N° 22_5452). Anonymised data may be made available upon reasonable request to the corresponding author.

## References

[1] Calabrese L.H., Calabrese C., Cappelli L.C. (2018). Rheumatic immune-related adverse events from cancer immunotherapy. Nat. Rev. Rheumatol..

[2] Bluestone J.A., Anderson M. (2020). Tolerance in the Age of Immunotherapy. N. Engl. J. Med..

[3] Abdel-Wahab N., Suarez-Almazor M.E. (2019). Frequency and distribution of various rheumatic disorders associated with checkpoint inhibitor therapy. Rheumatology.

[4] Roberts J., Ennis D., Hudson M., Ye C., Saltman A., Himmel M., Rottapel R., Pope J., Hoa S., Tisseverasinghe A. (2020). Rheumatic immune-related adverse events associated with cancer immunotherapy: A nationwide multi-center cohort. Autoimmun. Rev..

[5] Braaten T.J., Brahmer J.R., Forde P.M., Le D., Lipson E.J., Naidoo J., Schollenberger M., Zheng L., Bingham C.O., Shah A.A. (2020). Immune checkpoint inhibitor-induced inflammatory arthritis persists after immunotherapy cessation. Ann. Rheum. Dis..

[6] Kostine M., Finckh A., Bingham C.O., Visser K., Leipe J., Schulze-Koops H., Choy E.H., Benesova K., Radstake T., Cope A.P. (2020). EULAR points to consider for the diagnosis and management of rheumatic immune-related adverse events due to cancer immunotherapy with checkpoint inhibitors. Ann. Rheum. Dis..

[7] Haanen J., Obeid M., Spain L., Carbonnel F., Wang Y., Robert C., Lyon A., Wick W., Kostine M., Peters S. (2022). Management of toxicities from immunotherapy: ESMO Clinical Practice Guideline for diagnosis, treatment and follow-up. Ann. Oncol..

[8] Cappelli L.C., Bingham C.O. (2021). Expert Perspective: Immune Checkpoint Inhibitors and Rheumatologic Complications. Arthritis Rheumatol..

[9] Draghi A., Borch T.H., Radic H.D., Chamberlain C.A., Gokuldass A., Svane I.M., Donia M. (2018). Differential effects of corticosteroids and anti-TNF on tumor-specific immune responses: Implications for the management of irAEs. Int. J. Cancer.

[10] Faje A.T., Lawrence D., Flaherty K., Freedman C., Fadden R., Rubin K., Cohen J., Sullivan R.J. (2018). High-dose glucocorticoids for the treatment of ipilimumab-induced hypophysitis is associated with reduced survival in patients with melanoma. Cancer.

[11] Arbour K.C., Mezquita L., Long N., Rizvi H., Auclin E., Ni A., Martínez-Bernal G., Ferrara R., Lai W.V., Hendriks L.E.L. (2018). Impact of Baseline Steroids on Efficacy of Programmed Cell Death-1 and Programmed Death-Ligand 1 Blockade in Patients With Non–Small-Cell Lung Cancer. J. Clin. Oncol..

[12] Li N., Zheng X., Gan J., Zhuo T., Li X., Yang C., Wu Y., Qin S. (2023). Effects of glucocorticoid use on survival of advanced non-small-cell lung cancer patients treated with immune checkpoint inhibitors. Chin. Med. J..

[13] Sebbag E., Lauper K., Molina-Collada J., Aletaha D., Askling J., Gente K., Bertheussen H., Bitoun S., Bolek E.C., Burmester G.R. (2025). 2024 EULAR points to consider on the initiation of targeted therapies in patients with inflammatory arthritis and a history of cancer. Ann. Rheum. Dis..

[14] De La Fuente F., Belkhir R., Henry J., Nguyen C.D., Pham T., Germain V., Gavand P.E., Labadie C., Briere C., Lauret A. (2022). Use of a bDMARD or tsDMARD for the management of inflammatory arthritis under checkpoint inhibitors: An observational study. RMD Open.

[15] (2006). Classification and Response Criteria Subcommittee of The American College of Rheumatology Committee on Quality Measures. Development of classification and response criteria for rheumatic diseases. Arthritis Care Res..

[16] US Department of Health and Human Services (2017). Common Terminology Criteria for Adverse Events (CTCAE), Version 5.0.

[17] Kostine M., Rouxel L., Barnetche T., Veillon R., Martin F., Dutriaux C., Dousset L., Pham-Ledard A., Prey S., Beylot-Barry M. (2018). Rheumatic disorders associated with immune checkpoint inhibitors in patients with cancer—Clinical aspects and relationship with tumour response: A single-centre prospective cohort study. Ann. Rheum. Dis..

[18] Calabrese C., Kirchner E., Kontzias K., Velcheti V., Calabrese L.H. (2017). Rheumatic immune-related adverse events of checkpoint therapy for cancer: Case series of a new nosological entity. RMD Open.

[19] Richter M.D., Crowson C., Kottschade L.A., Finnes H.D., Markovic S.N., Thanarajasingam U. (2019). Rheumatic Syndromes Associated With Immune Checkpoint Inhibitors: A Single-Center Cohort of Sixty-One Patients. Arthritis Rheumatol..

[20] Hysa E., Casabella A., Iandolino N., Gotelli E., Genova C., Tanda E.T., Pizzorni C., Smith V., Sulli A., Cutolo M. (2025). Clinical outcomes in cancer patients with immune checkpoint inhibitor-induced arthritis treated with methotrexate: A retrospective longitudinal monocentric pilot study. Clin. Exp. Rheumatol..

[21] Zhang S., Chen S.-D., Chen L., Hong B. (2025). Impact of glucocorticoid administration on therapeutic outcomes of immune checkpoint inhibitors in non-small cell lung cancer: A systematic review and meta-analysis. Front. Med..

[22] Zhang Y., Chen J., Liu H., Dai J., Zhao J., Zhu S., Zhang X., Liang J., Hu X., Zhao J. (2024). The incidence of immune-related adverse events (irAEs) and their association with clinical outcomes in advanced renal cell carcinoma and urothelial carcinoma patients treated with immune checkpoint inhibitors: A systematic review and meta-analysis. Cancer Treat. Rev..

[23] van Not O.J., Verheijden R.J., Eertwegh A.J.M.v.D., Haanen J.B.A.G., Aarts M.J.B., Berkmortel F.W.P.J.v.D., Blank C.U., Boers-Sonderen M.J., de Groot J.-W.B., Hospers G.A.P. (2022). Association of Immune-Related Adverse Event Management With Survival in Patients With Advanced Melanoma. JAMA Oncol..

[24] Bass A.R., Abdel-Wahab N., Reid P.D., Sparks J.A., Calabrese C., Jannat-Khah D.P., Ghosh N., Rajesh D., Aude C.A., Gedmintas L. (2023). Comparative safety and effectiveness of TNF inhibitors, IL6 inhibitors and methotrexate for the treatment of immune checkpoint inhibitor-associated arthritis. Ann. Rheum. Dis..

[25] Chen A.Y., Wolchok J.D., Bass A.R. (2021). TNF in the era of immune checkpoint inhibitors: Friend or foe?. Nat. Rev. Rheumatol..

[26] Bertrand F., Montfort A., Marcheteau E., Imbert C., Gilhodes J., Filleron T., Rochaix P., Andrieu-Abadie N., Levade T., Meyer N. (2017). TNFα blockade overcomes resistance to anti-PD-1 in experimental melanoma. Nat. Commun..

[27] Perez-Ruiz E., Minute L., Otano I., Alvarez M., Ochoa M.C., Belsue V., De Andrea C., Rodriguez-Ruiz M.E., Perez-Gracia J.L., Marquez-Rodas I. (2019). Prophylactic TNF blockade uncouples efficacy and toxicity in dual CTLA-4 and PD-1 immunotherapy. Nature.

[28] Montfort A., Filleron T., Virazels M., Dufau C., Milhès J., Pagès C., Olivier P., Ayyoub M., Mounier M., Lusque A. (2021). Combining Nivolumab and Ipilimumab with Infliximab or Certolizumab in Patients with Advanced Melanoma: First Results of a Phase Ib Clinical Trial. Clin. Cancer Res..

[29] Holmstroem R.B., Nielsen O.H., Jacobsen S., Riis L.B., Theile S., Bjerrum J.T., Vilmann P., Johansen J.S., Boisen M.K., Eefsen R.H.L. (2022). COLAR: Open-label clinical study of IL-6 blockade with tocilizumab for the treatment of immune checkpoint inhibitor-induced colitis and arthritis. J. Immunother. Cancer.

[30] Campochiaro C., Farina N., Tomelleri A., Ferrara R., Lazzari C., De Luca G., Bulotta A., Signorelli D., Palmisano A., Vignale D. (2021). Tocilizumab for the treatment of immune-related adverse events: A systematic literature review and a multicentre case series. Eur. J. Intern. Med..

[31] Sebbag E., Molina-Collada J., Ndoye R., Aletaha D., Askling J., Gente K., Bertheussen H., Bitoun S., Bolek E.C., Buch M.H. (2025). Systematic literature review and meta-analysis informing the EULAR points to consider on the initiation of targeted therapies in patients with inflammatory arthritis and a history of cancer. Ann. Rheum. Dis..

